# Inducing ferroptosis has the potential to overcome therapy resistance in breast cancer

**DOI:** 10.3389/fimmu.2022.1038225

**Published:** 2022-11-24

**Authors:** Xiaowen Qi, Zhixing Wan, Baohong Jiang, Yuhan Ouyang, Wenjie Feng, Hongbo Zhu, Yeru Tan, Rongfang He, Liming Xie, Yuehua Li

**Affiliations:** ^1^ Department of Medical Oncology, The First Affiliated Hospital, Hengyang Medical School, University of South China, Hengyang, Hunan, China; ^2^ Department of Pharmacy, The First Affiliated Hospital, Hengyang Medical School, University of South China, Hengyang, Hunan, China; ^3^ Department of Pathology, The First Affiliated Hospital, Hengyang Medical School, University of South China, Hengyang, Hunan, China; ^4^ Institute of Pathogenic Biology, Hengyang Medical College, University of South China, Hengyang, China

**Keywords:** ferroptosis, breast cancer, therapy resistance, autophagy, chemotherapy, radiotherapy, nanoparticles

## Abstract

Breast cancer is the most common type of malignancy among women. Due to the iron-dependent character of breast cancer cells, they are more sensitive to ferroptosis compared to normal cells. It is possible to reverse tumor resistance by inducing ferroptosis in breast cancer cells, thereby improving tumor treatment outcomes. Ferroptosis is highly dependent on the balance of oxidative and antioxidant status. When ferroptosis occurs, intracellular iron levels are significantly increased, leading to increased membrane lipid peroxidation and ultimately triggering ferroptosis. Ferroptotic death is a form of autophagy-associated cell death. Synergistic use of nanoparticle-loaded ferroptosis-inducer with radiotherapy and chemotherapy achieves more significant tumor suppression and inhibits the growth of breast cancer by targeting cancer tissues, enhancing the sensitivity of cells to drugs, reducing the drug resistance of cancer cells and the toxicity of drugs. In this review, we present the current status of breast cancer and the mechanisms of ferroptosis. It is hopeful for us to realize effective treatment of breast cancer through targeted ferroptosis.

## 1 Introduction

Breat cancer is the most prevalent malignancy among women (1). The current status of breast cancer treatment remains suboptimal, mainly using surgery, radiotherapy, chemotherapy, and targeted therapy. Drug resistance remains a major obstacle for clinicians in the treatment of breast cancer (2). Ferroptosis was first proposed by Dixon, S.J in 2012, is a novel form of cell death induced by erastin and RSL3, distinct from apoptosis, autophagy and necrosis, is an iron-dependent chain reaction of destructive membrane lipid peroxidation, which leads to an imbalance of intracellular redox state (3). Altered cellular redox status has an intimate relationship with malignant transformation and metastasis of cancer cells (4, 5).

Ferroptosis is associated with many cancer types, including breast cancer (6), lung cancer (7)and pancreatic cancer (8). Effective evasion of regulated cell death is one of the most important features of cancer. It has been found that cancer cells that have evaded other forms of cell death still maintain sensitivity to ferroptosis. It seems that induction of ferroptosis in breast cells has the potential to affect tumor drug resistance (9). Tumor stem cells are highly iron-dependent and have an important role in promoting tumor cell proliferation and invasion, which are the main causes of tumor recurrence and metastasis. These cells are insensitive to conventional anticancer therapy, but can induce ferroptosis by modulating iron metabolism to exert more effective antitumor effects (10). Combined use of ferroptosis inducers during cancer radiotherapy and chemotherapy can effectively promote the sensitivity of cancer cells to ferroptosis and considerably improve the effectiveness of tumor treatment (11, 12).

Induction of ferroptosis in breast cancer cells can significantly inhibit tumor cell growth (13, 14). In breast cancer cells, the expression of transferrin receptor1 (TFR1), certain six transmembrane epithelial antigen of the prostate (STEAP) family members and Hepcidin were upregulated, while the expression of ferroportin (FPN) was downregulated. This suggests that breast cancer cells are iron-dependent and more sensitive to ferroptosis inducers (15). Long-chain acyl-coenzyme A synthetase 4 (ACSL4) is participated in lipid peroxidation formation and presents a high expression in a subpopulation of triple-negative breast cancer (TNBC) cell lines. The expression of ACSL4 positively correlates with breast cancer cell ferroptosis sensitivity (16). Glutathione (GSH) deficiency is associated with malignant transformation of breast cancer cells (17). Thioredoxin reductase 1 protein (TXNRD1), glutathione pathway and superoxide dismutase are predominantly and commonly regulated in breast cancer. High thioredoxin expression is strongly related to increased oxidative stress and poor prognosis in breast cancer. Cells with TXNRD1 knockdown (KO)are more sensitive to ferroptosis (18). GTP Cyclohydrolase (GCH) expression is associated with tumor development as well as angiogenesis. Upregulation of GCH1 expression in breast cancer cells stimulates proliferation and growth of cancer cells, results in poor prognosis of breast cancer. The use of GCH1 inhibitors suppress tumor growth and induce a switch in tumor immune response from M2 to M1 polarization of tumor associated macrophages. M2 is associated with tumor angiogenesis and metastasis (19, 20). Inhibition of GCH1 activity increases the susceptibility of drug-resistant cancer cells to ferroptosis (20, 21). Nuclear factor erythroid 2-related factor 2 (NRF2) exerts its antioxidant effects by upregulating the expression of genes related to iron and ROS metabolism and HO-1 to reduce ROS levels, increasing chemoresistance and ferroptosis resistance in breast cancer cells. The upregulated expression of heme oxygenase -1 (HO-1) in breast cancers has an inhibitory effect on cancer cell proliferation and invasion, and displays a dual role in ferroptotic cells, which depends on intracellular oxidative stress levels (22–24).

Ferroptosis offers a new direction in the treatment of breast cancer, but how to avoid its side effects is still an open question. In the presents of ferroptosis activation carries with the risk of inducing neurodegenerative disease and exacerbating ischemia-reperfusion injury (25–28). Ferroptotic damage also includes inflammatory reactions such as inflammatory bowel disease (29) and acute pancreatitis (30). In-depth understanding of ferroptosis metabolism in breast cancer is of utmost importance in searching for new breast cancer therapeutic-agents.

## 2 Current status of breast cancer and its treatment

Breast cancer is a major public health problem that threatens women’s health and is the most prevalent malignancy among women (1). Women in the 50-64 age group are at high risk of breast cancer, and the prevalence is significantly higher in women than in men, with only about 1% of breast cancers occurring in men (31, 32). An epidemiological survey on breast cancer shows that the development of breast cancer is mainly affected by estrogen levels, with about 10% of breast cancers are associated with genetic mutations (1). Long-term exposure to estrogen, obesity, smoking, alcohol consumption, previous history of radiation therapy to the chest, and increased breast density can all increase the risk of breast cancer. Proper exercise can reduce the risk of breast cancer (1). With the improvement of medical technology as well as the early detection and interventional treatment of breast cancer, the incidence and effective cure rate of breast cancer have increased in recent years. The U.S. Preventive Services Task Force recommends that women aged 50-74 have a mammogram every two years to improve breast cancer screening rates (1, 31). Breast cancer has a distinct tumor heterogeneity, with multiple subtypes and differences in incidence, treatment options and prognosis for each subtype (33). Breast cancer can be classified into four molecular subtypes based on the expression of Estrogen receptor (ER), Progesterone receptor (PR) and human epidermal growth factor receptor type 2 (HER-2) with the use of immunohistochemistry: luminal A, luminal B, HER-2 and TNBC (34). Current treatments for breast cancer mainly use surgery, radiotherapy, chemotherapy and targeted therapy, but a single treatment method does not achieve the expected therapeutic effect, and a combination of surgery-based treatment with other means is usually adopted (34). Breast cancer, with its many subtypes, is mainly treated with surgery and chemotherapy, supplemented by treatments based on the specificity of each subtype of receptor. Such as endocrine therapy for ER and PR receptor-positive breast cancer patients and anti-HER-2+ therapy for HER-2 positive breast cancer patients. As the most refractory type of breast cancer (33, 34), TNBC can be divided into four subtypes base on the heterogeneity of molecular characteristics, metabolomics and tumor microenvironment (35–38), which including mesenchymal-like (MES), luminal androgen receptor (LAR), basal-like and immune-activated (BLIA), basal-like and immune-suppressed (BLIS) subtypes (39, 40). Most of the current clinical trials focus on LAR in TNBC, applying an AR antagonist alone (41, 42) or in combination with a phosphatidylinositol 3-kinase (PI3K) inhibitor (43, 44), or combining immunotherapy to achieve AR inhibition with immune checkpoint blockade (45). The use of CDK4/6 inhibitors and hormone therapy in luminal B patients provides a strategy for the treatment of breast cancer without chemotherapy (46). The combination of PI3K inhibitors with aromatase inhibitors can produce positive effects, but their toxic effects are not negligible (47). HER-2-positive breast cancer patients will benefit from the dual inhibitory effect of trastuzumab and lapatinib on HER-2 (48). Estrogen receptor-positive breast cancer cells are highly susceptible to PI3K mutations, making the combination of letrozole and taselisib more effective (49). Nanoparticle albumin-bound paclitaxel (nab-Paclitaxel) in luminal A reduced the toxicity and increased the antitumor activity of paclitaxel (50). We have summarized the molecular subtype-based emerging clinical trials for breast cancer in **Table 1**. LAR tumors have higher fatty acid metabolic activity, ROS levels and overexpression of lipoxygenase than the other three subtypes of TNBC, all of which are evidence that LAR tumors are more vulnerable to ferroptosis (40, 51). High expression of CD44 in mesenchymal state tumor cells activates iron metabolic pathways, resulting in increased cellular susceptibility to ferroptosis (52). BLIA and BLIS were less correlated with ferroptosis (40). The combination of GPX4 inhibitors with immune checkpoint inhibitors for ferroptosis induction and enhanced immunosuppression has great potential for LAR tumor therapy (40). Discovering and developing safer and more effective drugs are warranted.

**Table 1:** Emerging therapies for breast cancer based on molecular subtypes.

Molecular subtypes	Drugs	Phase	Target
HER-2 +	trastuzumab and lapatinib	II	HER2 blockade (48)
Luminal A	Etrozole and taselisib	II	ER and PI3K (49)
	Nanoparticle albumin-bound paclitaxel	II	B tubulin (50)
Luminal B	Ribociclib and letrozole	II	CDK4/6 and hormone receptor (46)
	Buparlisib and capecitabine	I	PI3K and aromatase (47)
TNBC	abiraterone acetate and prednisone	II	AR and PI3K (43)
	Bicalutamide	II	AR (41)
	Enzalutamide	IB/II	AR and PI3K (44)
	GT0918	I	AR (42)
	Pembrolizumab and Enobosarm	II	AR and programmed death receptor (PD-1) (45)

Breast cancer patients have a high rate of drug toxicity, drug resistance and recurrence during treatment (2). Tumor cell genomic instability is the main cause of tumor heterogeneity, which drives the evolution of cancer cells, affects their sensitivity to therapeutic agents, and ultimately promotes tumor drug resistance (53–55). Tumor drug resistance is also associated with the available concentration of drugs in the tumor as well as the tumor microenvironment (54). The tumor microenvironment involves complex interactions between cancer cells and stromal cells. Alterations in the tumor microenvironment can lead to changes in the properties of stromal cells and their secretion of soluble small molecules, which can lead to microenvironmental mediating tumor drug resistance (56). Ferroptosis exerts anti-tumor effects by engaging complicated crosstalk between tumor cells and immune cells to mediate tumor immunity (57, 58). KRAS is the key to macrophage polarization and its alteration leads to tumor associated macrophages formation and M2-like pro-tumor phenotype (59). A tumor-associated macrophage type is associated with immunosuppression (19). CD8+ T cells promote tumor cell ferroptosis and induce radiosensitization *via* IFN (60).

## 3 Mechanism of ferroptosis

Catalyzed by iron and iron-dependent enzymes, cells produce functional oxidative metabolites and promote labile iron pool (LIP) formation, while inevitably leading to the accumulation of some undesirable oxidative byproducts (61–63). When they accumulate to a lethal level can cause severe cellular damage and even lead to cell death. Therefore, antioxidant mechanisms have evolved in cells to remove these metabolic wastes in a timely manner, such as the glutathione peroxidase-4-GSH (GPX4-GSH) system, Coenzyme Q10 (CoQ10) (64).

Ferroptosis is an iron-dependent form of regulated cell death (3), characterized by massive accumulation of disruptive membrane lipid peroxidation (65). There are three main features of ferroptosis including imbalance of iron metabolism, massive production of lipid peroxides, and collapse of the GPX4-GSH system (58). Morphologically, ferroptotic cells show significant changes in mitochondrial morphology, with mitochondrial contraction, rupture of the outer mitochondrial membrane (OMM), and enlarged mitochondrial cristae. In the absence of swelling or contraction of cells in necrosis and apoptosis, neither nuclear changes nor chromatin condensation (3, 66, 67).

**Figure 1:**
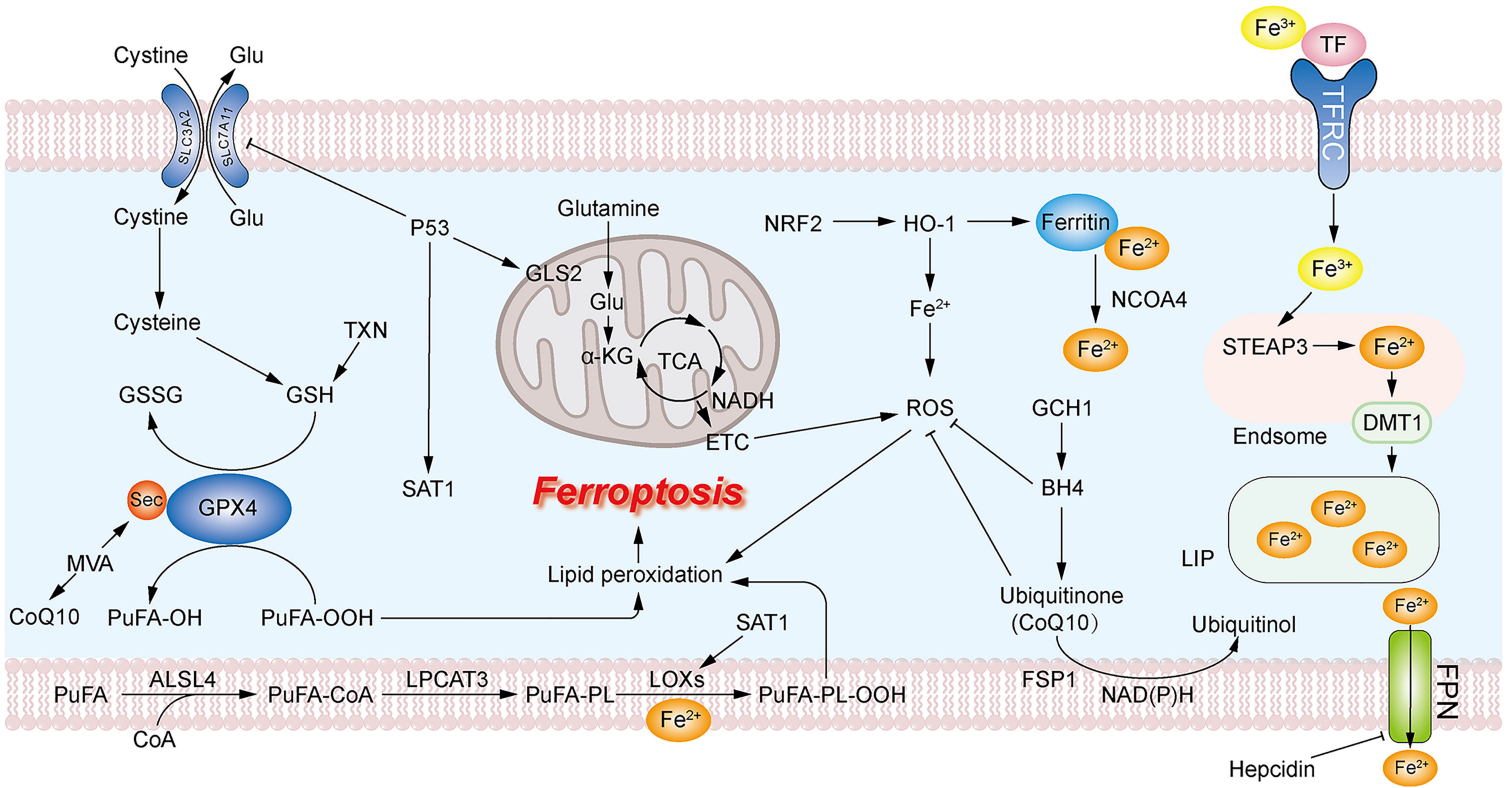
Mechanism of ferroptosis. The massive accumulation of PUFA on the cell membrane leads to excessive production of PUFA-OOH. GPX4 uses GSH as a reducing agent to reduce PUFA-OOH to PUFA-OH, reducing the production of lipid peroxides. GSH can be formed from cystine transported into the cell *via* system XC- or *via* the TXN pathway. The accumulation of intracellular ROS promotes lipid peroxidation. Mitochondrial GLS2 promotes glutamine catabolism to facilitate ROS production leading to the accumulation of lipid peroxides. Large accumulation of intracellular ferrous ions leads to overproduction of ROS. CoQ10 acts as an antioxidant to inhibit ROS production, and the MVA pathway and GCH1-BH4 are associated with the production of CoQ10.

Lipid peroxidation due to massive accumulation of the iron positively regulate ferroptosis, while GSH depletion due to system xc- and GPX4 inactivation negatively regulate ferroptosis (61–63). Apart from the classical GPX4-GSH axis, there are other antioxidant mechanisms involved in the negative regulation of ferroptosis in breast cancer cells, such as the ferroptosis suppressor protein 1-NADH-CoQ10 (FSP1-NADH-CoQ10) axis (68) and the GCH1 -Tetrahydrobiopterin (GCH1-BH4) axis through the involvement of COQ10 (69), and the regulation of some antioxidant transcription factors, such as NRF2 (70). A mutant of p53 can promote ferroptosis (71, 72). Here, we summarized and mapped the ferroptosis mechanism in **Figure 1**. FINs in breast cancer are summarized in **Table 2**.

**Table 2:** FINS in breast cancer.

Target	Drugs
Increased iron	Sulfasalazine
	Lapatinib+siramsine
	Neratinib
	Artemisinin
Mitochondrial disorders	RF-A
	Nitroxide
Reduced iNOS activity	GA
Inactivation of GPX4	DT
	Metformin
	Simvastatin
	Curcumin
Inhibition of GSH synthesis	Metformin
	Sulfasalazine
	BSO+AUR
Inhibition of CoQ10 synthesis	FIN56

### 3.1 Intracellular iron metabolism and its redox reactions

Iron is one of the most important trace elements in the human body and essential for the vital activities of the body, participating in the formation and regulation of the activity of Reactive Oxygen Species (ROS)-producing enzymes, such as Lipid oxidases (LOXs) (3). Iron homeostasis plays a key role in controlling the balance between ROS production and ROS scavenging as well as cellular redox and potential oxidative damage (73, 74). Elevated iron levels in mitochondria may lead to excessive production of ROS (65). High iron diet causes ferroptosis in mouse cardiomyocytes (75). Iron levels are significantly elevated in ferroptosis cells, suggesting that the accumulation of intracellular iron is a prerequisite for cells to undergo ferroptosis. Deferoxamine (DFO) inhibits erastin-induced cell death by chelating intracellular iron and reducing iron overload (3). Transferrin (TF) and TFR1 regulate ferroptosis by mediating cellular uptake of iron (26). Nuclear receptor coactivator 4 (NCOA4) regulates ferroptosis by mediating ferritinophagy to control iron homeostasis (76). Ferritin consists of ferritin heavy chain (FTH) and ferritin light chain (FTL), of which FTH1 has iron oxidase activity and oxidizes ferrous ion to ferric ion (77). Knockdown or inhibition of FTH1 both promote ferroptotic death (76, 78).

Sulfasalazine targeting Transferrin receptor (TFRC) and its ferroptosis-inducing effect is reduced in ER-positive breast cancer (14). The combination of lapatinib and siramsine induces ferroptosis in breast cancer cells rather their individual treatment. Promoting the expression of transferrin and degradation of FPN, causing a time-dependent increase in intracellular iron levels and ROS levels, ultimately leading to cellular ferroptosis and autophagy at different time (79). Neratinib causes iron imbalance by regulating the expression of proteins related to the iron transport system, ultimately inducing ferroptosis (80). Artemisinin mediates the degradation of ferritin, which is an elevated level of intracellular ferrous iron, leading to cellular ferroptosis (81).

Ferric ions from foods bind to TF in blood and attach to TFR1 on the cell membrane, transporting ferric ions into the cell, where STEAP3 in acidic nuclear endosome reduces ferric ions to ferrous ions. Ferrous ions are transferred *via* divalent mental transporter 1 (DMT1) to LIP. Binding to ferritin is the storage form of intracellular free irons, and NCOA4 is involved in the degradation of ferritin, releasing ferrous ions (76).

Excess Ferrous ions generate large amounts of hydroxyl radicals through Fenton and Haberweth reactions, which alter the intracellular redox state. Due to their high instability and reactivity, hydroxyl radicals can cause severe damage to lipids and proteins and intense oxidative damage to DNA (82, 83). In ferroptotic cells, hydroxyl radicals are able to attack polyunsaturated fatty acids (PUFAs) on membranes, triggering membrane lipid peroxidation (9).

### 3.2 Mitochondrial involvement in reactive superoxide formation

Mitochondria is important for maintaining normal cellular function, energy supply and redox homeostasis, and is a major site for intracellular ROS production. The Tricarboxylic Acid (TCA) cycle and electron transport chain (ETC) action are necessary for mitochondria to produce sufficient ROS *via* oxidative phosphorylation (OXPHOS) (84, 85). Ferroptotic cells undergo significant changes in mitochondrial morphology with mitochondrial contraction, rupture of the OMM, and enlarged mitochondrial cristae (3, 67). Robustaflavone A (RF-A) promotes Voltage-dependent anion-selective channel protein 2 (VDAC2) expression and ubiquitinated degradation, inducing the breakdown of mitochondrial functional systems, lipid peroxidation and ROS production, ultimately leading to ferroptosis in breast cancer cells. Blocking mitochondrial function contributes to ferroptosis inhibition independent of GPX4 activity (86). Nitroxide targets mitochondria as a ROS scavenger and inhibits lipid peroxidation of mitochondrial membranes thereby inhibiting ferroptosis (87). BAY87-2243 induces ferroptosis in melanoma cells through inhibition of mitochondrial respiratory chain complex 1 and induction of mitochondrial membrane potential depolarization (88). In cysteine-starved cells, mitochondrial metabolism is significantly enhanced, promoting GSH depletion, ROS production and ferroptosis. Mitochondrial GLS2 promotes glutamine catabolism to glutamate, which is then converted to α-KG *via* glutamine dehydrogenase into the TCA cycle, driving ETC and leading to mitochondrial membrane hyperpolarization and lipid peroxide accumulation, eventually ferroptosis is triggered (26, 85).

### 3.3 Accumulation of lipid peroxides

PUFAs are major components of membrane lipids, which are highly susceptible to oxidation and play an important role in maintaining membrane integrity and participating in trans-plasma membrane transport activity (89). Increased production of lipid peroxides occurs in both erastin- and RSL-induced ferroptosis (90). Extensive production of ROS attack PUFAs on the membrane, triggering membrane lipid peroxidation, leading to a massive accumulation of lipid peroxides and ferroptosis (9). The presence of more and longer PUFAs exacerbates ferroptosis (91). Glycyrrhetinic acid (GA) generates ROS and Reactive nitrogen species (RNS) by upregulating NADPH oxidase and iNOS activity in TNBC cells, which exacerbates intracellular oxidative stress level, leads to lipid peroxidation and ferroptosis (92). Inhibition of peroxidation of PUFAs with antioxidants inhibits ferroptoisis (3). Acyl coenzyme A (CoA) synthase ACSL4 and lysophosphatidylcholine acyltransferase 3 (LPCAT3) are involved in the synthesis of lipid ROS and their deletion contributes to ferroptosis resistance (93). ACSL4 is engaged in the production and activation of the long-chain polyunsaturated fatty acids arachidonic acid (AA) and adrenalic acid (AdA), acylating PUFA to form PUFA-CoA (16), LPCAT3 inserts PUFA-CoA into membrane phospholipids (PL) and catalyzes the production of PUFA-PL (94). Finally, PUFAs are oxidized by iron and iron-dependent oxidase LOXs to produce PUFA-PL-OOH, which initiates ferroptosis (95).Oxidized PUFAs accumulate on the membrane, causing membrane thinning and bending to increase the accessibility of oxidants. Oxidants react with PUFAs in the membrane, forming a positive feedback loop, which further accelerates membrane instability and ultimately leads to irreversible damage to membrane integrity and promotes cellular ferroptosis (96).

### 3.4 XC-/GPX4-GSH system

GSH is vital in normal embryonic growth and development as well as an essential reducer. GPX4 catalyzes the reduction of harmful lipid peroxides to harmless lipid alcohols, thus protecting cell membranes from peroxidative damage by PUFA-OOH (17, 97, 98). Cystine-starved cells with reduced GSH synthesis are more sensitive to ferroptosis inducers (FINs) (26). Overexpression of SLC3A1 enhances tumor progression in breast cancer cells, while blocking SLC3A1 with specific siRNA or SLC3A1-specific inhibitor sulfasaliazine inhibits tumor growth (99). Erastin acts on system xc- to inhibit cystine uptake, and intracellular GSH synthesis is depressed (3). RSL inactivates GPX4 by binding to selenocysteine, with massive accumulation of lipid peroxides (100). When GPX4 is inhibited or knocked down, intracellular antioxidant activity is significantly diminished and lipid peroxides accumulate excessively, eventually leading to ferroptosis (101, 102). Sulfasaliazine induces ferroptosis in breast cancer cells by functioning on system xc- which is currently in a type II clinical trial. Dihydroisotanshinone I (DT) induces cellular ferroptosis and inhibits tumor growth without adverse effects through down-regulation of GPX4 expression (103). Metformin enhanced ferroptosis in breast cancer cells by altering the stability of SLC7A11, downregulating GPX4 activity and inhibiting the autophagy induced by H19. This effect is more sensitive in estrogen receptor-positive breast cancer cells. In addition, the combination of metformin with sulfasazine enhanced its ferroptosis induction and exerted more effective anti-cancer effects (104–106). MVA pathway and the activity of GPX4 is inhibited in ferroptotic death breast cancer cells induced by Simvastatin (107). Curcumin induces ferroptosis in breast cancer cells by upregulating the expression of redox target genes such as HO-1 and downregulating antioxidants such as GPX4, an effect that is more pronounced than in normal human breast epithelial cells (108). Moreover, high expression of Glycogen synthase kinase-3β (GSK-3β) was able to increase the sensitivity of erastin-induced ferroptosis by enhancing the inhibition of GPX4 (109).

System xc- is a glutamate-cystine transporter located on the plasma membrane, consisting of the heavy chain subunit SLC3A2/CD98hc and the light chain subunit xCT/SLC7A11, responsible for cellular uptake of cystine and transport of glutamate (110). Cystine is reduced to cysteine upon entry into the cell, then glutamate-cysteine ligase (Gcl) and glutathione synthetase (Gss) catalyze the production of GSH (111, 112). GSH is the most abundant and common antioxidant in cells, maintaining intracellular redox homeostasis. GPX4 is one of the glutathione peroxidases, selenocysteine is an important component of the GPX4 active center (113). Mevalonate is involved in the synthesis of selenoprotein in the GPX4 active center (114). GPX4 uses GSH as an essential reducing agent to catalyze the reduction of harmful lipid peroxides to harmless lipid alcohols, thereby protecting cell membranes from peroxidative damage by PUFAs. When the function of system xc- is inhibited, TXN pathway can be an alternative GSH synthesis pathway. TXNRD1 KO cell survival is highly dependent on intracellular GSH levels (115). Buthionine sulfoximine (BSO) can induce cell death in TXNRD1 KO cell (116). Forced expression of xCT in cells which are completely deficient in GSH production, TXN pathway increases cellular cystine uptake to rescue GSH deficiency. Overexpression of xCT in TXNRD1 KO cells not only exacerbates but also accelerates BSO-induced cell death (117). Expression of TXNRD1 are higher in Gclm(-/-) mice compared to WT mice (118). Thus, the TXN pathway is another major antioxidant approach that has been shown to support cell survival after system xc- inhibition, TXN and system xc- synergistically control intracellular GSH level (119). Due to the presence of the TXN pathway and the essential role of GPX4 in the embryo, directly targeting of GPX4 is more effective than inhibiting the activity of SLC7A11 when inducing ferroptosis (98, 120). BSO can induce ferroptosis by inhibiting GCL and thus decreasing GSH synthesis (121). However, inhibition of GSH by BSO alone can only elevate ROS at the tumor initiation stage and cannot affect established tumor growth (17). Auranofin (AUR) is an FDA-approved thioredoxin reductase inhibitor for the suppression of TNBC tumor growth (122). Combining BSO with AUR can significantly increase the mortality of breast cancer cells through combined inhibition of GSH synthesis and TXN pathway (17).

### 3.5 COQ10 as an endogenous membrane antioxidant inhibits ferroptosis

CoQ10 is involved in respiratory chain activities in the mitochondrial membrane and is critical for electron translocation. The non-mitochondrial CoQ10 acts as a free radical trapping antioxidant (RTA) and prevents plasma membrane lipid damage. MVA pathway is engaged in CoQ10 skeleton generation (114, 123). A significant decrease in CoQ10 level occurs in ferroptotic cells (124). The MVA pathway is involved in CoQ10 backbone formation, and FIN56 induces ferroptosis by reducing CoQ10 production *via* the MVA pathway (125). Inhibition of CoQ10 synthesis by inhibiting CoQ10 synthase CoQ2 increases RLS-induced lipid ROS and exacerbates ferroptosis (126). CoQ10 is involved in ferroptosis resistance through the FSP1-NADH-CoQ10 axis and the GCH1-BH4 axis.

#### 3.5.1 FSP1-NADH-CoQ10

FSP1 is the key component of the antioxidant system in ferroptotic death independent of the GPX4-GSH axis (68). FSP1 expression positively correlates with cancer cell resistance to ferroptosis induced by GSH depletion or GPX4 inhibition (98, 123). FSP1 KO leads to increased cellular phospholipid oxidation and increased sensitivity to ferroptosis inducers. NAD(P)H-quinone oxidoreductase-1 (NQO1) is a CoQ oxidoreductase that may be involved in CoQ10 reduction in synergy with FSP1 to regulate ferroptosis (123). NQO1 knockdown cells showed increased sensitivity to erastin- and sorafenib-induced ferroptosis (78). Elevated NADH/NADPH ratio indicates a weakened intracellular antioxidant capacity and a greater susceptibility to cellular ferroptosis (127). FSP1 targets the plasma membrane and converts oxidized CoQ10 (ubiquitinone) to reduced CoQ10 (ubiquitinol), NAD(P)H acts as a reducing co-substrate to provide hydrogen ions for this reaction, which inhibits lipid peroxidation and ferroptosis (68, 123).

#### 3.5.2 GCH1-BH4-phospholipid axis

The GCH1-BH4-phospholipid axis links to ferroptosis resistance. GTP Cyclohydrolase 1 (GCH1) is the key enzyme that catalyzes the production of tetrahydrobiopterin (BH4) (69). The expression level of GCH1 determines the BH4 availability, which influence the redox balance in cancer cell. Intracellular levels of BH4 are negatively correlated with oxidized GSH and NADP (128). An increase in BH4 can lead to an increase in CoQ10 levels. Inhibition of GCH1 activity results in the sensitivity of drug-resistant cancer cells to ferroptosis. Conversely, overexpression of GCH1 effectively prevents cell death induced by deletion of RSL3, IKE and GPX4, and inhibits lipid peroxidation (69). BH4 can act directly as an antioxidant or indirectly by synthesizing CoQ10 to inhibit lipid peroxidation and attenuate oxidative damage in the presence of FSP1, protecting cells from ferroptosis (69, 128).

### 3.6 NRF2 involved Redox homeostasis

NRF2 is a major antioxidant transcription factor *in vivo*. NRF2 increases cellular resistance to ferroptosis, by upregulating the expression of iron, HO-1, and ROS metabolism-related gene. The expression of NRF2 was upregulated in ferroptosis, while knockdown or pharmacological inhibition of NRF2 revealed the phenomena of GSH depletion, increased iron level and lipid ROS production in erastin- and sorafenib-induced cells, promoting cellular ferroptosis and enhancing the anticancer activity (70). Moreover, there is a p62-Keap1-NRF2 pathway to regulate intracellular NRF2 levels. P62 expression positively correlates with NRF2 levels, while Keap1 negatively regulates NRF2 and mediates its degradation (78). Kelch-like ECH-associated protein 1 (Keap1) binds to Cul3 and Rbx1 to form a functional E3 ubiquitin ligase complex that ubiquitinates NRF2 for degradation. This process can be inhibited by the NRF2-dependent transcriptional chemoattractants Sulforaphane and quinone (tBHQ)-induced oxidative stress, mainly because they enable a redox-dependent alteration of multiple cysteine residues in Keap1, and NRF2 separates from Keap1 and enters the nucleus (129, 130). In nucleus, NRF2 forms a heterodimer with sMaf (131) and binds to ARE (132), protecting cancer cells from GPX4 inhibition and promoting the transcription of antioxidant enzymes, such as HMOX1, NOQ1, and GSTS (108, 133–135), reducing ROS levels, forming resistance to ferroptosis (78).

HO-1 has been shown to have anti-proliferative, antioxidant and anti-inflammatory effects. Upregulated HO-1 expression in breast cancer cells has an inhibitory effect on cancer cell proliferation and invasion (23, 136). HO-1 degrades heme to CO, ferrous ions as well as bilirubin and can induce upregulation of ferritin expression. Ferritin binds to free intracellular iron and inhibits the Fenton reaction, thereby reducing ROS production and exerting its antioxidant activity (24). HO-1 acts as an antioxidant, and HO-1 expression is upregulated in erastin- and sorafenib-induced ferroptosis. Meanwhile, inhibition of HO-1 expression or the occurrence of HO-1 deficiency exacerbates intracellular ferroptosis (22, 78). However, HO-1, a major source of intracellular iron. Under high oxidative stress, a significant rise in intracellular concentration of ferrous ions, which increases ROS levels to promote lipid peroxidation and thus lead to ferroptosis (108, 137, 138). HO-1 is important for maintaining redox homeostasis and its dual role in ferroptosis may be related to intracellular levels of oxidative stress and cellular stress. In response to induction of cellular stress, HO-1 expression is moderate upregulated and acts as an antioxidant defense mechanism to mitigate ferroptosis. In contrast, when excessive intracellular oxidative stress occurs, HOs are overactivated and overexpressed, which acts as pro-oxidant to accelerate cellular ferroptosis (22, 137). The role of HO-1 in ferroptosis remains controversial, and the mechanism underlying its role in ferroptosis remains to be identified.

### 3.7 P53-mediated GSH synthesis and depletion

P53 is the most frequent and susceptible gene to mutation in breast cancer. In previous studies, it has been shown that mutant p53 has a higher mortality rate and worse prognosis than wild-type p53 (139–141). Induced restoration of the wild-type properties of mutant p53 offers a new idea for the treatment of breast cancer, and PRIMA-1MET(APR-246, Aprea AB) may be able to achieve this goal (71, 72). PRIMA-1MET increased intracellular GSH depletion and induced ROS production. Synergy with BSO increased the sensitivity of cells to PRIMA-1METm (142, 143). PRIMA-1MET can induce ferroptosis in AML cells (144). In addition, a novel ferroptosis inducer, MMRI62, with dual targeting of FTH1 and mutant p53, which induces ferroptosis in pancreatic cancer cells by inducing lysosomal degradation of FTH1 and NCOA4 as well as proteasomal degradation of mutant p53 to improve chemoresistance and control metastasis of cancer cells (145).

A P533KR (K117R + K161R + K162R) mutant, which fails to induce cell cycle arrest, senescence and apoptosis, but presence of inhibitory properties on SLC7A11 expression renders the cell incapable of cystine uptake, reduction in GSH synthesis, more susceptible to ferroptosis and can be inhibited by Fer-1. While overexpression of SLC7A11 in P533KR mutant rescues its ferroptosis. Suggesting that P53 triggers ferroptosis by mediating transcriptional repression of SLC7A11 (146). P534KR (K98R + K117R + K161R + K162R) mutant, with complete depletion of acetylation capacity compared to p533KR resulted in loss of ferroptosis induction, suggesting p53-mediated acetylation capacity plays an important role in ferroptosis induction (147). In addition, p533KR retains the transcriptional activity of glutaminase 2 (GLS2), which induces ferroptosis by promoting glutaminolysis (26, 147). P53 upregulates the expression of spermidine/spermine N1-acetyltransferase 1 (SAT1) and promotes ALOX15 activity, leading to lipid peroxidation (148). Mutant p53 also increases ferroptosis sensitivity of pancreatic cancer cells by downregulating the expression of FTH1 and NCOA4 (145).On the other hand, P53 inhibits ferroptosis by upregulating the expression of cell cycle protein-dependent kinase inhibitor 1A (CDKN1A/p21) (149) and GLS2 (26, 150), which also inhibit the formation of DPP4-NOX1 complex (151) by altering the localization and activity of DPP4 in CRC cells.

## 4 Correlation between autophagy and ferroptosis

Ferroptosis has an autophagic correlation (59, 76, 152, 153). Autophagy (Macroautophagy) is a form of cell death that exists within normal cells to maintain a state of intracellular homeostasis. It is a lysosomal degradation process that cells engulf cytoplasmic material to form autophagosomes, which then bind to lysosomes to form autolysosomes (154). Autophagic lysosomes can degrade protein, lipid and damaged mitochondria, etc. (152, 155, 156).

Elevated autophagic activity occurred in erastin-induced ferroptosis cells, whereas the use of the lysosomal inhibitors Bafa1 and CQ blocked ferroptotic death cells (76, 153), and this inhibition was time-differentiated, with a more pronounced inhibition effect at 12h than 24h. Autophagy genes (ATGs) were found to be involved in the positive regulation of ferroptosis by RNAi screening (76). Knockdown of ATGs or pharmacological inhibition both achieved the blocking effect of ferroptosis (76, 157).

**Figure 2:**
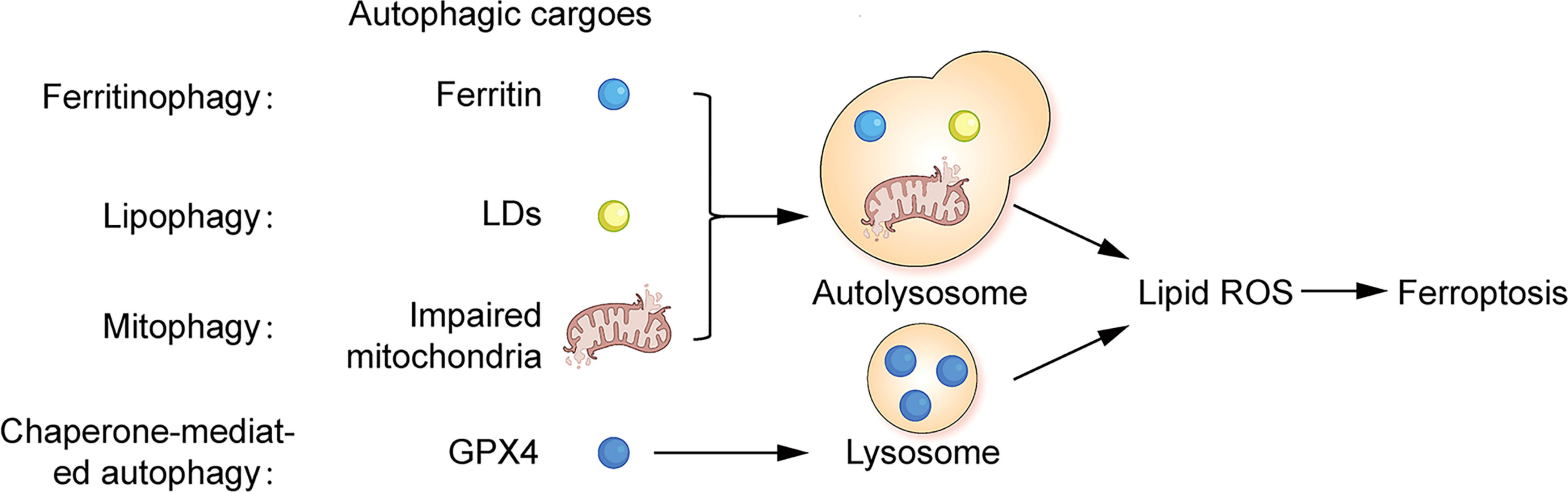
Correlation between ferroptosis and autophagy Ferritin, lipid droplets and impaired mitochondria can act as autophagic substrates for ferritinophagy, lipophagy, and mitophagy, forming autolysosomes in cells. GPX4 can act as autophagic substrates for CMA-mediated autophagy, forming lysosomes in cells. Facilitating the production of intracellular lipid ROS, leading to ferroptosis.

Ferritinophagy is a NCOA4-mediated ferritin degradation exists in ferroptotic death cells, which is an elevation of ferrous ions thereby promoting the accumulation of lipid ROS, while independent of GSH depletion (76, 153). Autophagic degradation of FTH1 is also found in erastin-induced cells (76). Lipophagy promotes ferroptosis by mediating the selective autophagic degradation of lipid droplet (LD). The accumulation of neutral LD protects cells from ferroptosis by suppressing lipid peroxidation (157). LDs are involved in the redistribution of PUFAs. PUFAs are transferred from the phospholipid membrane to the core of LDs. Where PUFAs are less susceptible to ROS attack, thus inhibiting lipid peroxidation (158). Mitophagy is a process of selective autophagic degradation of damaged or redundant mitochondria to maintain intracellular mitochondrial homeostasis (155). There is a mitochondrial autophagy-associated ferroptosis in BAY87-2243-induced human melanoma cells, which exerts an inhibitory effect on tumor growth (88). Chaperone-mediated autophagy (CMA) is a cellular autophagic degradation pathway that recognizes soluble cytoplasmic proteins containing specific KEFRQ motifs through heat shock-associated proteins (HSP) and targets them directly to the lysosome for degradation (155). HSP90 upregulates the level of lysosome-associated membrane protein type 2a (Lamp-2a), promotes chaperone-mediated autophagic degradation of GPX4, and thus participates in the regulation of ferroptosis (159, 160). A graphical representation of the relationship between ferroptosis and autophagy is shown in **Figure 2**.

Perhaps induction of cellular autophagy could be an effective way to activate ferroptosis (76).

## 5 Effective ways to enhance ferroptosis

In the process of breast cancer chemotherapy and radiotherapy, it is difficult to distinguish between normal cells and cancer cells. Thus it is hard to target breast cancer cells, which will inevitably cause damage to normal tissues and lead to high toxicity and adverse effects (2). The discovery of ferroptosis emerges a new ray of light for cancer treatment. However, the poor water solubility and rapid metabolism of therapeutic drugs lead to their low bioavailability *in vivo*. Nanoparticles have the characteristics of small size and low toxicity, ferroptosis-inducers (FINs) can be loaded on these particles, which can help us solve these problems. Nano-FINs in breast cancer cells are summarized in **Table 3**. More importantly nanoparticles can target drug transport to tumor cells, reducing the toxic damage effect of drugs on normal cells. The application of Nanoparticle-loaded ferroptosis-inducer-targeted transport technology can greatly enhance the tumor suppressive effect of the drugs (161). Drug resistance of cancer cells is also a thorny issue in current cancer treatment. FINs can be used as chemotherapy and radiotherapy sensitizers enhance ferroptosis of cancer cells, achieving effective improvement in drug resistance during treatment and prolong patients’ survival (11, 12).

**Table 3:** Nano-FINS in breast cancer.

Nanomedicines	Composition	Cell
TA-Fe/ART@ZIF	artemisinin +tannic acid+Fe(II)+zeolitic imidazolate framework-8	MDA-MB-231
Fe3O4@PCBMA	simvastatin+Fe3O4+zwitterionic polymer coated magnetic nanoparticles	MDA-MB-231+MCF-7
erastin@FA‐exo	Folate+Erastin+Exosome	MDA-MB-231
HMCM	MnO2+HMCu2-xS+Nanoparticles	MCF-7
DFTA	Doxorubicin+FeCl3+tannic acid	MCF7

### 5.1 Nanomaterial-based therapeutic drugs

TA-Fe/ART@ZIF, a ferrous nanocarrier encapsulated with ART enhanced the ferroptosis-inducing effect of ART in TNBC cells and exhibited stronger tumor suppression compared to ART alone (162). A novel nanomedicine Fe3O4@PCBMA-SIM can slow down the metabolism of the drug and increase the accumulation and duration of action at the tumor site to exert better cancer suppressive effects (107). A newly discovered folate (FA)-exosome-encapsulated erastin can help us address the low water solubility and nephrotoxicity of erastin and target erastin delivery to FA receptor overexpressing TNBC cells. This erastin@FA-exo induced ferroptosis by inhibiting the expression of GPX4, upregulating the expression of cysteine dioxygenase (CDO1), and increasing the depletion of GSH is intracellular production of excess ROS, which greatly enhanced the antitumor effect of erastin (163). A heparanase (HPSE)-driven sequential released nanoparticles, NLC/H(D + F + S) NPs, induces ferroptosis characterized by excessive ROS production and GSH depletion in mouse breast cancer cells, significantly inhibits the metastatic growth of tumors and improves anti-cancer efficiency (164).

### 5.2 Ferroptosis inducers as sensitizers for chemotherapy

A new nanomedicine DFHHP can inhibit tumor growth by inducing apoptosis and ferroptosis in tumor cells to overcome tumor chemoresistance. DFHHP is an integration of Fe (VI) species and Doxorubicin (DOX) into HMS nanomaterials. DOX, a common chemotherapeutic agent in tumor therapy, generates a large amount of reactive superoxide radicals by promoting tumor cell reoxidation. DFHHP provides exogenous iron, generating highly reactive ROS by Fenton reaction, leading to the depletion of GSH and exacerbating ferroptosis of tumor cells (11). HMCM nanocomposites have photothermal properties that enable PT, and the nanodrug also incorporates the autophagy promoter Rapa, which enhances the sensitivity of breast cancer cells to ferroptosis and effectively controls tumor growth (165).

A drug-organics-inorganics self-assembled nanosystem (DFTA) effectively inhibits the progression of ER+ breast cancer by using a chemotherapeutic agent DOX, a ferroptosis inducer ferric chloride (FeCl3) and a activator of superoxide dismutase (SOD) tannic acid (TA), which activates a cascade reaction generated by intracellular ROS and significantly reduces GSH levels. In addition the combination with photothermal therapy (PT) can greatly increase the efficiency of ROS production. It is expected to achieve the combination of chemotherapy, PT, and ferroptosis against ER+ breast cancer (166).

### 5.3 Ferroptosis inducers as sensitizers for radiotherapy

Radiotherapy mainly uses targeted delivery of ionizing radiation (IR) to cause cell death. Hypoxia is the main mechanism leading to radiotherapy resistance in tumor cells, while hypoxia-induced ROS production and massive activation of the hypoxia-inducible factors result in the induction of ferroptosis (12, 167). The use of FINs may overcome hypoxia-induced resistance to radiotherapy by promoting ferroptosis in tumor cells. Conversely, inhibition of ferroptosis leads to resistance to radiotherapy (12).

Tumor cells treated with radiotherapy showed typical ferroptotic features, with mitochondrial atrophy and its increased membrane density, enhanced lipid peroxidation, as well as increased expression of the ferroptosis marker gene prostaglandin-endoperoxide synthase-2 (PTGS2) (12). IR can promote the production of PUFA-PLs by upregulating the expression of ACSL4, while stimulating cells to produce large amounts of ROS, leading to lipid peroxidation and inducing ferroptosis in cancer cells (12, 168). Meanwhile IR may inhibit ferroptosis by inducing the expression of SLC7A11 and GPX4 as a negative feedback regulatory pathway to induce radiotherapy resistance in cancer cells. The combination of sulfasalazine, an ferroptosis inducer targeting SLC7A11, and IR enhanced the sensitivity of cancer cells to radiotherapy, synergistically induced ferroptosis, and significantly inhibited tumor growth (120, 169). Another study found that IR also antagonized the upregulation of SLC7A11 expression by activating P53, making cancer cells more sensitive to ferroptosis. The combination of FINs and radiation therapy is more effective in the treatment of P53-mutated cancers (170). CD8+ T cells promote tumor cell ferroptosis and induce radiosensitization *via* IFN. Immunotherapy-activated CD8+T cells induce tumor cell ferroptotic death by producing IFN in concert with radiotherapy -activated ATM targeting SLC7A11 to inhibit cystine uptake (60). Immunotherapy enhances the efficacy of radiotherapy, radiation and immunotherapy synergistically induce ferroptosis in tumor cells (60, 171).

## 6 Discussion

Ferroptosis is an iron-dependent form of lipid peroxidative cell death. With GSH as a reducing agent and CoQ10 as an endogenous membrane antioxidant to inhibit lipid peroxidation and ferroptosis (28, 69). Mitochondria are involved in ferroptosis by promoting glutaminolysis (26, 85). NRF2 and P53 have dual roles in ferroptotic cells. Whether CoQ10 could be a new target for ferroptosis? What is the role of HO-1 in ferroptosis and how does it work? Nevertheless, in-depth studies are required to clarify the mechanism of ferroptosis. However, it is clear that induction of ferroptosis in breast cancer cells inhibits tumor growth (13, 14). Given the positive role of autophagy in facilitating ferroptosis, perhaps autophagy activation can be used as a target to induce cellular ferroptosis (76, 153). FINs can be used as sensitizers for radiotherapy and chemotherapy to enhance tumor efficacy (11, 12). We are expected to realize the combination of nano-ferroptosis-inducers with chemotherapy and radiotherapy. It can not only enhance the targeting effect of drugs, but also solve the problem of drug resistance and greatly promote the tumor suppression effect. However, the toxic side effects associated with this treatment modality are elusive and require further investigation. It is imperative to develop new ferroptosis-inducing drugs that are highly effective and less toxic. In summary, induction of ferroptosis has the potential to surmount treatment resistance in breast cancer.

## Author contributions

XWQ and YHL conceived the content. ZXW ,WJF and BHJ collected all data. YHO, HBZ and RYT analysed and sorted the data. WJF,YHY XWQ are response to pictureS. LMX and YHL were the major contributors in writing and modified the manuscript. All authors read and approved the final manuscript.

## Funding

This study was supported by the National Natural Science Foundation of China (No.81902707, YL), the Key Research Project of Hunan Provincial Education Department (21A0270, YL), China Postdoctoral Science Foundation (2022M711541), YL the Natural Science Foundation of Hunan Province (2019JJ80036, RH, 2021SK51818, LX) and Scientific Research Fund Project of Hunan Provincial Health Commission (B202303109577, D202303109450, 20201974, B20180052).

## Conflict of interest

The authors declare that the research was conducted in the absence of any commercial or financial relationships that could be construed as a potential conflict of interest.

## Publisher’s note

All claims expressed in this article are solely those of the authors and do not necessarily represent those of their affiliated organizations, or those of the publisher, the editors and the reviewers. Any product that may be evaluated in this article, or claim that may be made by its manufacturer, is not guaranteed or endorsed by the publisher.

## Glossary

**Table:**
